# The effect of terminal globular domains on the response of recombinant mini-spidroins to fiber spinning triggers

**DOI:** 10.1038/s41598-020-67703-1

**Published:** 2020-06-30

**Authors:** William Finnigan, Aled D. Roberts, Cosimo Ligorio, Nigel S. Scrutton, Rainer Breitling, Jonny J. Blaker, Eriko Takano

**Affiliations:** 1 Department of Chemistry, Manchester Institute of Biotechnology, Manchester Synthetic Biology Research Centre SYNBIOCHEM, The University of Manchester, Manchester, M1 7DN UK; 2 Department of Materials, Manchester Institute of Biotechnology, The University of Manchester, Manchester, M1 7DN UK; 3 Bio-Active Materials Group, Department of Materials, The University of Manchester, Manchester, M13 9PL UK

**Keywords:** Industrial microbiology, Biomaterials

## Abstract

Spider silk spidroins consist of long repetitive protein strands, flanked by globular terminal domains. The globular domains are often omitted in recombinant spidroins, but are thought to be essential for the spiders’ natural spinning process. Mimicking this spinning process could be an essential step towards producing strong synthetic spider silk. Here we describe the production of a range of mini-spidroins with both terminal domains, and characterize their response to a number of biomimetic spinning triggers. Our results suggest that mini-spidroins which are able to form protein micelles due to the addition of both terminal domains exhibit shear-thinning, a property which native spidroins also show. Furthermore, our data also suggest that a pH drop alone is insufficient to trigger assembly in a wet-spinning process, and must be combined with salting-out for effective fiber formation. With these insights, we applied these assembly triggers for relatively biomimetic wet spinning. This work adds to the foundation of literature for developing improved biomimetic spinning techniques, which ought to result in synthetic silk that more closely approximates the unique properties of native spider silk.

## Introduction

Spider dragline silk has impressive mechanical properties, with high strength and good extensibility resulting in a level of toughness which exceeds all other natural and synthetic fibers^1^. However, unlike silkworms, spiders cannot be efficiently farmed for their silk^2^. For this reason, the production of recombinant spider silk proteins (spidroins), and their subsequent spinning into synthetic spider silk fibers, has been an active topic of research for a number of decades^3–7^.

**Figure 1:**
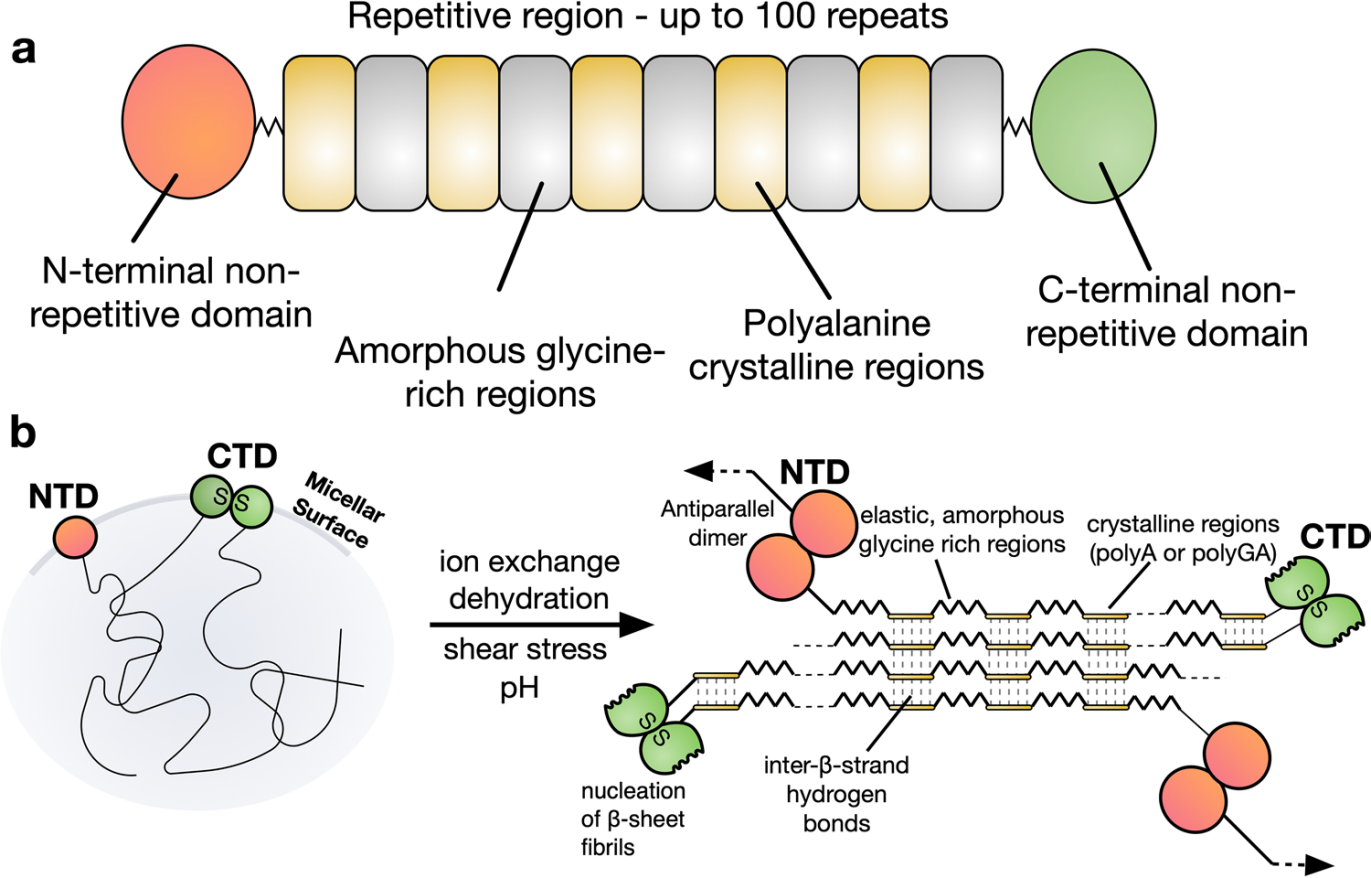
Schematic representation of a native spider silk protein (**a**) The domain structure of spider silk proteins, consisting of non-repetitive N- and C-terminal domains, flanking a much larger repetitive section, which alternates between glycine-rich regions and polyalanines. (**b**) A model for the conversion of soluble spidroins, stored as protein micelles, into insoluble silk fibers through the assembly triggers of shearing force or elongational flow, changing pH, dehydration and changing salts in the silk gland of a spider. Reproduced from Ref.^72^.

Major ampullate spider silk proteins (spidroins) are typically 200–350 kDa in size and constitute the dragline silk of spiders. Generally, spidroins have three distinct regions (Fig. 1)^3^. The vast majority of the protein is repetitive, consisting of alternating polyalanine regions and glycine-rich regions^8^. At the terminals of the spidroin exist non-repetitive domains, referred to as the N- and C-terminal domains (NTD and CTD). These globular terminal domains are crucial in facilitating the soluble storage of the spidroins at high concentrations (30–50% w/v) in the silk gland, and in initiating fiber assembly^9^.

Much of the research into recombinant spider silk has focused on spidroins consisting of only the repetitive region. Whilst some of the largest recombinant spidroins have resulted in fibers with good mechanical properties, larger repetitive regions typically result in poor spidroin yields^10,11^. Commonly, denaturing conditions have been employed in either the purification or spinning processes. Silk proteins purified under such denaturing conditions have been shown to lack the response to shear exhibited by native spinning dopes—an essential feature to provide alignment of the fiber microstructure, as well as an assembly trigger itself^12^. In contrast, biomimetic spinning utilising correctly folded terminal domains may offer the production of relatively biomimetic spinning dopes, and a route to synthetic spider silk fibers with superior mechanical properties^3,9^.

In spiders, dragline spidroins are stored in an ampulla (or sac) in the silk gland, where the highly concentrated spinning dope forms a lyotropic liquid crystalline solution^13,14^. Upon spinning, the spidroins proceed through a long and increasingly narrow S-shaped spinning duct where the coordinated action of acidification, ion exchange, dehydration, shearing force and elongational flow, is proposed to trigger assembly and promote alignment of β-sheet nanocrystals as the fibers are formed (Fig. 1)^5,14,15^. Chaotropic sodium and chloride ions are replaced with potassium and kosmotropic phosphate ions during the spinning process, inducing salting out of the spidroins^16–18^. Chaotropic ions have been shown to prevent intra- and intermolecular interactions on the recombinant repetitive regions, while kosmotropic ions promote hydrogen bond interactions in the glycine-rich regions^19^. The pH drops from pH 7.6 at the beginning of the duct, to pH 5.7 by halfway through, and likely lower near the spinneret, as a result of the action of a carbonic anhydrase^20,21^. The decreasing pH causes conformational changes in the N- and C-terminal domains, which act as regulatory elements for the control of spidroin assembly and ensure solubility during storage in the ampulla^3,22^. In contrast, the molecular structure of some recombinant repetitive regions have been shown not to respond to pH^23^.

The NTD is known as the ‘lock’, as this domain dimerises in response to the decreasing pH^24,25^. This dimerization ‘locks’ the spidroins into an infinite network, as the CTDs form a disulphide-linked dimer^26–28^. The CTD is proposed to partially unfold in response to decreasing pH. This change is thought to cause the CTD to form β-sheet amyloid fibrils, nucleating the formation of β-sheet fibrils in the repetitive region, in a process analogous to the nucleation of various kinds of amyloid fibers^26,27,29^.

Recent work demonstrated that a small mini-spidroin featuring both terminal domains could be spun into a fiber by wet-spinning using a coagulation bath at pH 5.0, rather than the more commonly used denaturing methanol or isopropanol^9,30^. Here we build upon this to further investigate the expression of a range of mini-spidroins featuring pH-responsive terminal domains, which we have termed “complete” mini-spidroins to differentiate them from mini-spidroins consisting of only a repetitive region. We demonstrate the effects of pH, ion exchange and shearing force, which spiders employ during spinning, on one of these complete mini-spidroins and identify potentially relevant triggers for the development of better biomimetic spinning techniques. Finally, we use the resulting understanding of these assembly triggers to biomimetically spin synthetic spider silk fibers.

## Results and discussion

### Functional terminal domains are necessary to produce complete mini-spidroins for biomimetic spinning

**Figure 2:**
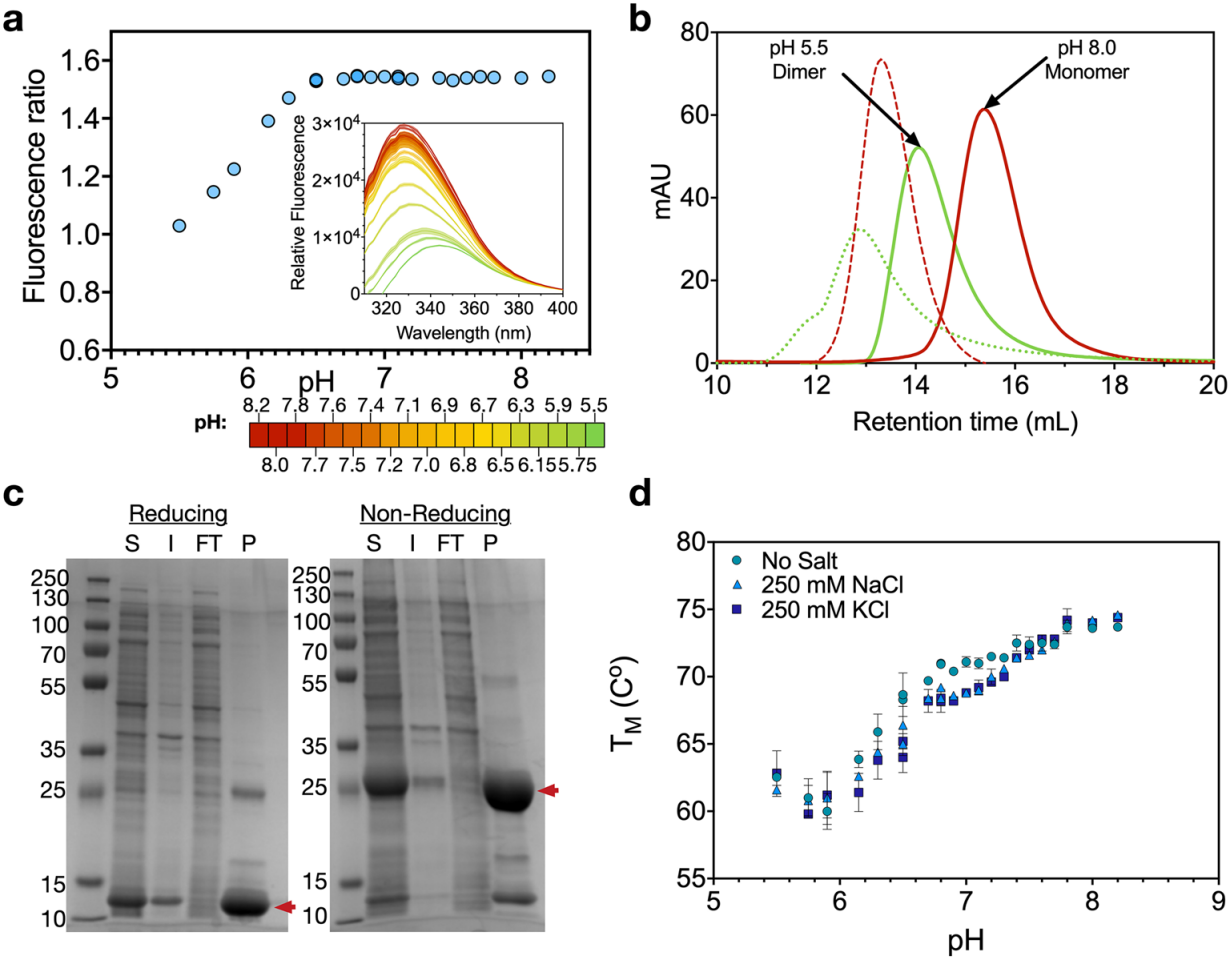
Characterisation of N- and C-terminal domains. (**a**) Tryptophan fluorescence of NTD2. The ratio between fluorescence at 338 and 352 nm as a function of pH is shown. The full fluorescence spectra at different pH values is included as an inset, with pH indicated by colour (red at pH 8.2 through to green at pH 5.5). Error bars show the standard deviation of three technical replicates. (**b**) Samples were run at pH 8 (red) and pH 5.5 (green), either in the presence of 300 mM NaCl (solid lines) or in its absence (dashed lines). With NaCl, peak elution corresponded to monomer and dimer species at pH 8 and pH 5.5, respectively. In the absence of NaCl, peaks were shifted to higher apparent molecular weights, possibly indicating the formation of larger multimers. Following elution, samples in the absence of salt appeared cloudy, suggesting aggregation. mAU stands for milli-absorbance units. (**c**) Two independent reducing and non-reducing SDS-PAGE gels showing the purification of CTD1 by nickel immobilized metal affinity chromatography. S: Soluble, I: Insoluble, FT: Flow-through, P: Purified. A band corresponding to a CTD1 monomer is observed under reducing conditions, while a band corresponding to a CTD1 dimer is observed under non-reducing conditions (red arrows). (**d**) Thermal stability of CTD1 with or without NaCl or KPi, as determined by dynamic scanning fluorometry (Supplementary Fig. S2). As pH decreases, there is a decrease in CTD1 thermal stability. Error bars show the standard deviation of three technical replicates.

We first sought to identify and characterize highly expressed, soluble and pH-responsive N- and C-terminal domains, which we selected from major ampullate spidroins 1 and 2 of *Latrodectus hesperus* (Supplementary Fig. S1, NTD1, NTD2, CTD1 and CTD2 for major ampullate spidroins 1 and 2 respectively. NTD2 and CTD1 were used for this study). A tryptophan residue, which is buried in the monomer of NTDs, has previously been shown to become exposed in the dimer conformation allowing the conformational change leading to dimerization to be followed by tryptophan fluorescence^31^. NTD2 showed a large shift in fluorescence with decreasing pH suggesting such a conformational change (Fig. 2a). Size exclusion chromatography (SEC) showed NTD2 to form a dimer at pH 5.0, while remaining a monomer at pH 8.0, in the presence of 300 mM NaCl (Fig. 2b). However, we noted that NTD2 eluted slightly later than expected, likely due to the addition of an unstructured section of repetitive region in the protein used in this experiment (Supplementary Fig. S1a), other experiments were carried out without this unstructured region. In the absence of NaCl, NTD2 eluted earlier from the SEC column. In addition, following the SEC in these conditions the samples appeared visibly cloudy.

CTD1 was confirmed to form a disulphide-linked dimer by non-reducing SDS-PAGE (Fig. 2c). A spidroin featuring both NTD2 and CTD1 is therefore expected to polymerise via end-to-end linking of the terminal domains, upon dimerization of NTD2. CTD1 was shown to become less thermostable with decreasing pH by dynamic scanning fluorimetry (Fig. 2d, Supplementary Fig. S2)^32–34^. Higher initial fluorescence was also observed in this assay with decreasing pH (Supplementary Fig. S3), indicating more exposed hydrophobic regions at lower pH values. These results correspond with CTD1 partially unfolding with decreasing pH, suggesting a sequence prone to hydrophobic β-aggregation present in CTD1 and other C-terminal domains^26,27^, consistent with the amyloid nucleation concept described above.

Having characterized suitable terminal domains, these were incorporated into a mini-spidroin expression vector—pTE1253, into which different repetitive regions could be easily cloned for the production of complete mini-spidroins. We adopted a cloning scheme utilising Type IIS restriction sites, allowing both pseudo-scarless duplication of repetitive regions, and their transfer into pTE1253 (Supplementary Fig. S4 and S5).

### Smaller complete mini-spidroins offer substantially higher protein yields

**Figure 3:**
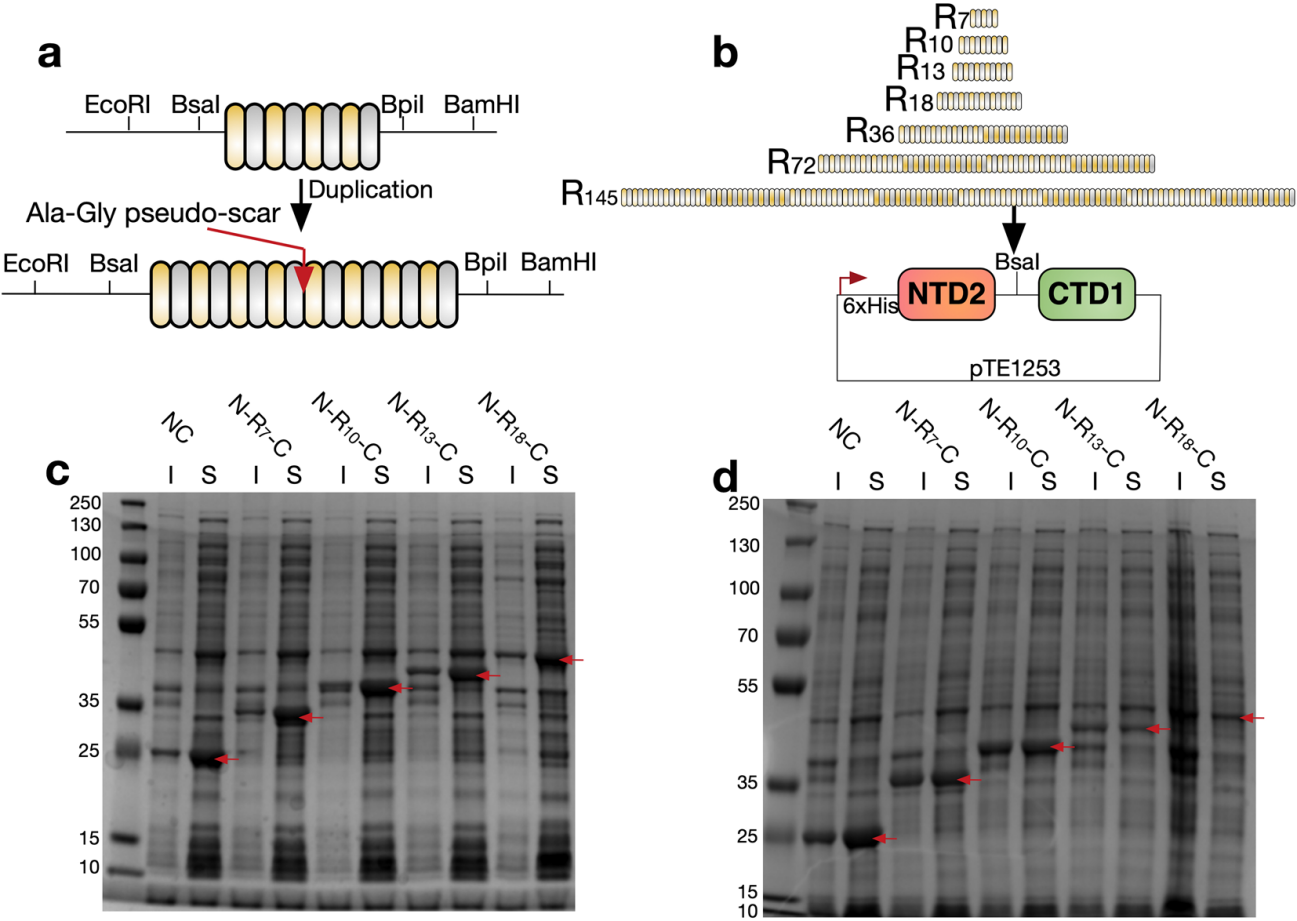
Heterologous expression of spidroins. Cloning scheme for (**a**) the duplication of repetitive regions, and (**b**) their incorporation into the pTE1253 vector for expression of complete mini-spidroins with both terminal domains. Both the duplication of repetitive regions, and their transfer into pTE1253 utilise type IIS restriction enzymes BsaI and BpiI resulting in an innocuous scar sequence which codes for alanine-glycine (Supplementary Fig. S4). Grey and yellow segments are used to illustrate alternating glycine-rich and polyalanine regions. Lighter and darker shading of these sections in B are used to highlight duplicated sequences. (**c**) and (**d**). SDS-PAGE of soluble (S) and insoluble (I) fractions of *E. coli* lysate following expression of various mini-spidroins for four hours (**c**) or 20 h (**d**) at 20 °C. Overexpressed proteins at the expected molecular weight are indicted by red arrows. Markers to the left of each gel are shown in kDa.

A range of different sized repetitive regions were generated from a codon-optimised DNA sequence for a section of the repetitive region of the major ampullate spidroin 1 from *Latrodectus hesperus.* Repetitive regions were cloned into pTE1253 to generate a range of “complete” mini-spidroins with both terminal domains (NTD2 and CTD1) (Fig. 3a,b). In addition, we generated a construct featuring both terminal domains but no repetitive region, designated NC throughout. Expression of these constructs in *E. coli* BL21(DE3) resulted in good levels of expression for constructs up to N-R_18_-C, at four hours post induction (Fig. 3c) compared to 20 h of incubation (Fig. 3d). Expression of constructs larger than this (N-R_36_-C to N-R_291_-C) were not detected by SDS-PAGE (Supplementary Fig. S6).

Smaller mini-spidroins allowed substantially higher levels of expressed soluble protein when cultures were grown overnight. In comparison, the expression level for the larger mini-spidroins decreased with longer growth times, likely due to intracellular aggregation (Supplementary Fig. S6). Indeed, mini-spidroins with larger repetitive regions are proposed to be more aggregation-prone^26^. Efforts to express the repetitive regions alone yielded no visible expression by SDS-PAGE analysis of *E. coli* lysates (data not shown).

Higher yields were obtained for the smaller mini-spidroins (~ 30 mg/L purified protein for N-R_18_-C, ~ 420 mg/L purified protein for N-R_7_-C), and all could be purified to high purity using nickel immobilized metal affinity chromatography (Supplementary Fig. S7). Following dialysis, small mini-spidrions N-R_7_-C and N-R_10_-C could be concentrated to at least 30% w/v as is seen in spiders^35^, without premature aggregation using centrifugal concentrators. During this process a more viscous phase was observed to form at the bottom of the concentrator, as compared with the rest of the solution. In contrast, processing of the larger mini-spidroin N-R_18_-C was not possible using a centrifugal concentrator due to aggregation. Others have used NaCl to help stabilise recombinant repetitive regions (without terminal domain), with good success concentrating these proteins^36^.

### A complete mini-spidroin featuring both terminal domains displays shear thinning

**Figure 4:**
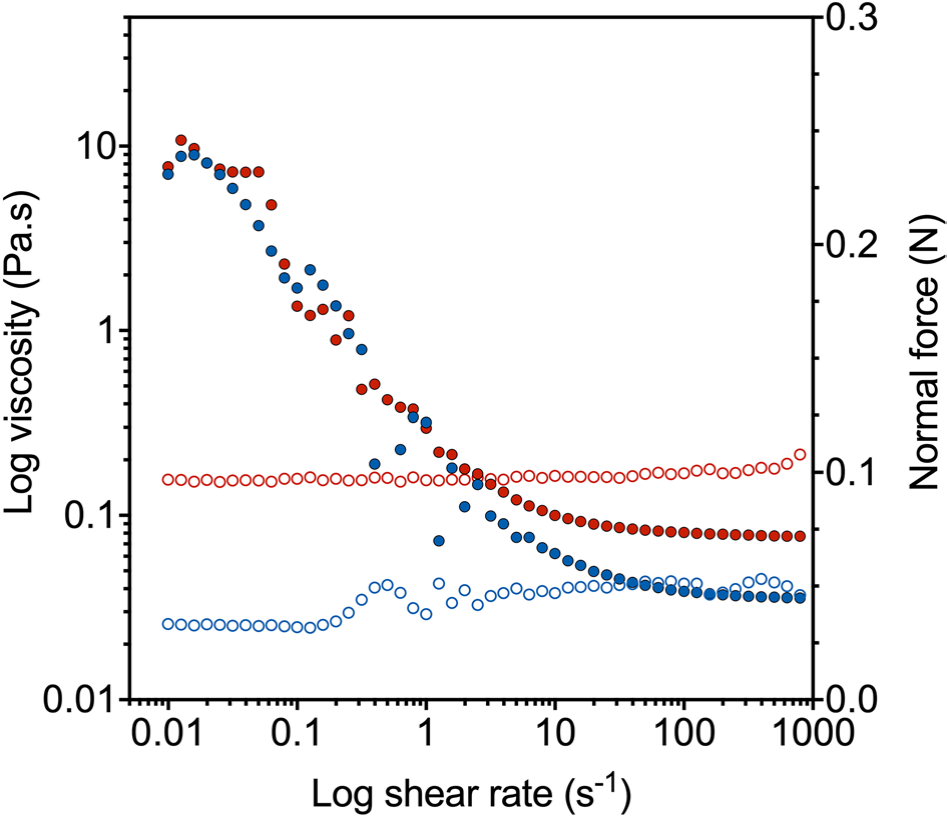
Viscosity of N-R7-C at 200 mg/mL (20% w/v) in response to shear. Two samples were tested with multiple consecutive runs. Shear history did not appear to have an effect on the rheology of the sample. Representative data is shown for each sample (sample A—red, sample B—blue). Closed circles show viscosity (Pa.s, left axis), open circles show normal force (N, right axis). Samples showed shear thinning behaviour, with no increase in normal force. All data is available in Supplementary Fig. S9.

The rheological properties of N-R_7_-C at 200 mg/mL (20% w/v) at pH 8.0 were investigated (Fig. 4, Supplementary Fig. S8 and S9). Data below γ = 0.01 s^-1^ could not be reliably collected and are not shown. Above a critical shear rate of γ_c_ ~ 0.02 s^-1^ the shear viscosity reduced with increasing shear rate, displaying shear-thinning behaviour. The data followed a power-law trend with η = K(γ)^b^, where η = viscosity, γ = shear rate and b = –0.77 ± 0.10 (n = 4, Fig. 4). This trend was similar to the typical shear-thinning and power-law trends observed for polymer melts^37–39^. Interestingly, the values of the exponent b were comparable with the data observed by Holland et al. for silkworm spinning dope, where b =  − 0.78 with n = 21 and standard deviation = 0.118^40^.

Concentrated small complete mini-spidroins similar to N-R_7_-C have been shown to form protein micelles in solution^9,30^. Recombinant spidroins featuring a spider eggcase silk repetitive region and both terminal domains have also been shown to form protein micelles^41^. Others discuss the formation of biomimetic spinning dopes (BSDs) from recombinant spidroins, with a suggestion that the formation of a BSD requires the inclusion of both terminal domains, resulting in protein micelles^13,42^. We propose that N-R_7_-C also forms protein micelles, and the observed shear thinning of N-R_7_-C is a result of the spidroin micelles aligning to the flow under shear. A parallel might be drawn with the alignment of red blood cells under flow, which are able to align to the blood flow at high shear rates due to deformability and orientation^43,44^. Interestingly, spidroins missing one or both terminal domains do not show similar behaviour, and indeed a repetitive region with only a CTD was shown to undergo shear thickening^45^.

It has previously been suggested that the zero-shear viscosity is an important value to take into account for spinnability, as this considers the intermolecular association and friction between silk molecules in the dope, and can therefore give an insight into how an applied energy can flow through the material^12^. Approximating our first data points as the zero-shear plateau region, two repeat samples (sample A and sample B) showed an initial viscosity of 9.0 ± 2.2 Pas, which is similar to the zero-shear plateau region for reconstituted silkworm silk at approximately the same concentration^12^. At high shear rates, our samples displayed Newtonian behaviour suggesting alignment of the spidroin micelles under shear, with a plateau of viscosities ranging from 0.037 ± 0.003 and 0.085 ± 0.011 Pa s for sample A and sample B, respectively (Fig. 4 and Supplementary Fig. S9). These values for infinite shear plateau viscosity are similar to values reported for reconstituted silkworm silk, where viscosities between 0.01 and 0.1 Pas were recorded for 4.5% and 18.5% dry weight concentration respectively^12^.

Our mini-spidroin showed no shear-thickening events, and multiple repeated runs did not result in a difference in rheological behaviour. Our data suggests shear does not act as an assembly trigger itself for our small mini-spidroin, despite this being a trigger for native spider silk dopes and other artificial spidroins^40^. We propose this is also partly due to the small size of N-R_7_-C. Indeed, this property likely facilitates their high expression levels (Fig. 3c), and their processing to a suitably high concentration as soluble protein.

Another important parameter to consider during viscosity measurements is represented by the change in the normal force generated during shear. In fact, a force perpendicular to shear is generated when a nonlinear response arises from molecules stretched under restraint, for example between the plates of a rheometer^46^. It has been proposed that when a silk dope is under shear, stretching of silk protein backbones can provide energy accumulation and consequent stress-induced phase transition^47^. For this reason, an increase in normal force could be used as an indicator of occurring a shear-induced transitions, such as conformational changes^48^, gelation^49^ and silk fibrillogenesis^46^, while a constant value of normal force can be attributed to simple alignment of the molecules under shear. As shown in Fig. 4, our mini-spidroins aligned to the flow as the shear rate increases, with no significant increase for the normal force, which remains constant at an average value of 0.082 ± 0.016 N. This result aligns with our hypothesis that our mini-spidroins aligned to the flow under shear, with no clear sign of a shear-induced phase transition.

The shear thinning observed can be regarded as a useful property from a manufacturing perspective, facilitating injection or wet spinning without issues of nozzle clotting or aggregation for other biopolymers and peptide systems^50^. Indeed, both native spider silk and silkworm spinning dopes display shear thinning^40^, where nozzle clotting could be lethal. Further work could look into shear-induced polarised light imaging (SIPLI) for further insight into other specific shear-induced phenomena, such as alignment and orientation as has been shown for other biopolymer and peptide systems^51,52^. Another interesting avenue of investigation would be the effect of different ions on the viscosity of N-R_7_-C. Calcium ions have been shown to form salt bridges between acidic amino acids in silkworm silk, resulting in feedstocks with higher viscosity. In contrast, monovalent ions such as potassium ions have been shown to screen this effect, resulting in a lower viscosity^53,54^. Determining whether the same effects would occur for N-R_7_-C will require further experimentation.

### Both a pH drop and salting out are required for effective biomimetic wet spinning

Recent work has shown a complete mini-spidroin similar to N-R_7_-C to be spinnable by biomimetic wet spinning into a pH 5.0 coagulation bath^9^. However, in the cited work, a high ionic strength solution was used to spin fibers for mechanical testing, although the effect of increasing ionic strength was not investigated. We were therefore interested in examining the effect of increasing ionic strength on the wet spinning of N-R_7_-C. Potassium phosphate was chosen to mimic the exchange of sodium and chloride ions for potassium and phosphate ions in the natural spinning process, which is proposed to induce salting out of the spidroins^16–18^, with kosmotropic ions promoting hydrogen bond interactions in the glycine-rich regions of the repetitive domains^19^.

**Figure 5:**
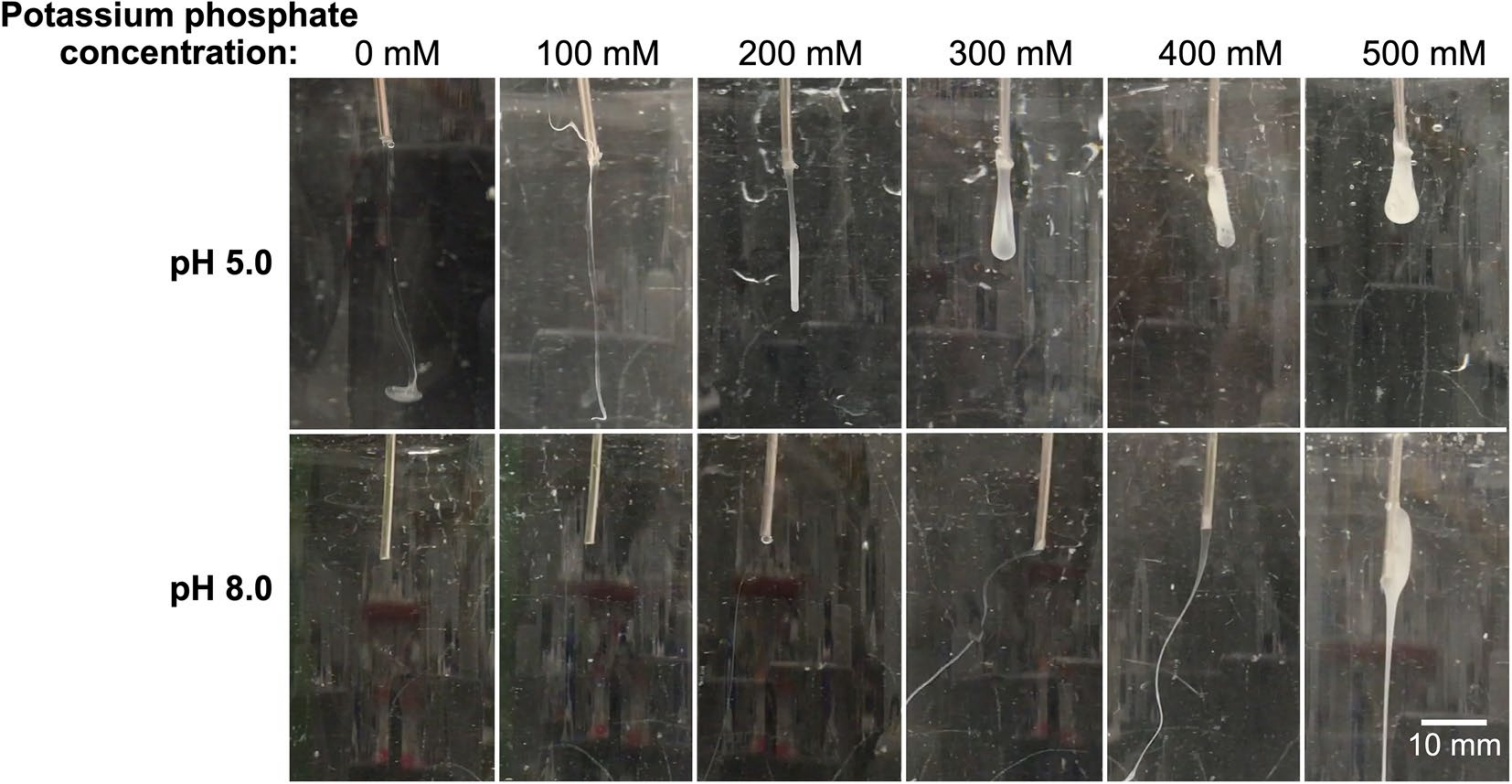
Wet spinning images of N-R_7_-C. Images show the effects of differing pH and potassium phosphate concentrations on the coagulation of N-R_7_-C at 100 mg/mL (10% w/v), as it is extruded through a blunted 16G needle at 25 µL/min. Potassium phosphate was prepared at the relevant pH by mixing solutions of KH_2_PO_4_ and K_2_HPO_4_ in each case. Approximate scale bar determined using the 16G needle diameter (1.6 mm) as a reference.

N-R_7_-C at 100 mg/mL was extruded via a needle into a range of buffer conditions at both pH 5.0 and pH 8.0, and the resulting fibers observed (Fig. 5). Potassium phosphate was prepared at the relevant pH by mixing solutions of KH_2_PO_4_ and K_2_HPO_4_. At pH 8.0, high (> 300 mM) potassium phosphate concentrations resulted in visible aggregates that appeared to extrude from the needle as a fiber, likely due to salting-out of the mini-spidroin. Collection of fibers at pH 8.0 at any KPi concentration was not possible, as they completely disintegrated upon contact in solution.

In contrast, at pH 5.0 with the addition of increasing concentrations of potassium phosphate, progressively more robust fibers formed. This resulted in an aggregated mass forming, rather than fibers, at higher potassium phosphate concentrations (> 300 mM). In the absence of potassium phosphate (0 mM), a colloidal suspension was observed. The use of 200 mM KPi or less at pH 5.0 resulted in what appeared visually to be fibers, but these were friable and could not be collected since they disintegrated when touched.

At higher KPi concentrations at pH 5.0, fibers could be collected or pulled and stretched from aggregates at the needle tip using tweezers (pH 5.0, > 300 mM KPi only). Based on these observations, we conclude that both a drop to pH 5.0 in combination with salting-out is necessary for the formation of robust fibers which can be collected, and either of these triggers alone is insufficient. Importantly, these conditions allow an artificial dope with suitable rheological properties to be formed, which can then be mechanically drawn into a fiber.

We also note that a requirement for salting out has been shown not to be necessary for the self-assembly of some native and recombinant spidroins^55–57^. Importantly, self-assembly is able to occur on a time-scale of hours, while fiber formation in a spinning process must occur over seconds, resulting in different requirements. Interestingly, potassium ions have been shown to screen the effects of salt bridges formed by metal ions such as calcium in silkworm spinning dopes^53,54^. Whilst there were no such metal ions present during the spinning trials carried out in this study, investigating these effects whilst spinning N-R_7_-C could offer further insight in future studies.

### Further investigation into the effects of pH and salt on the domains of N-R_7_-C

**Figure 6:**
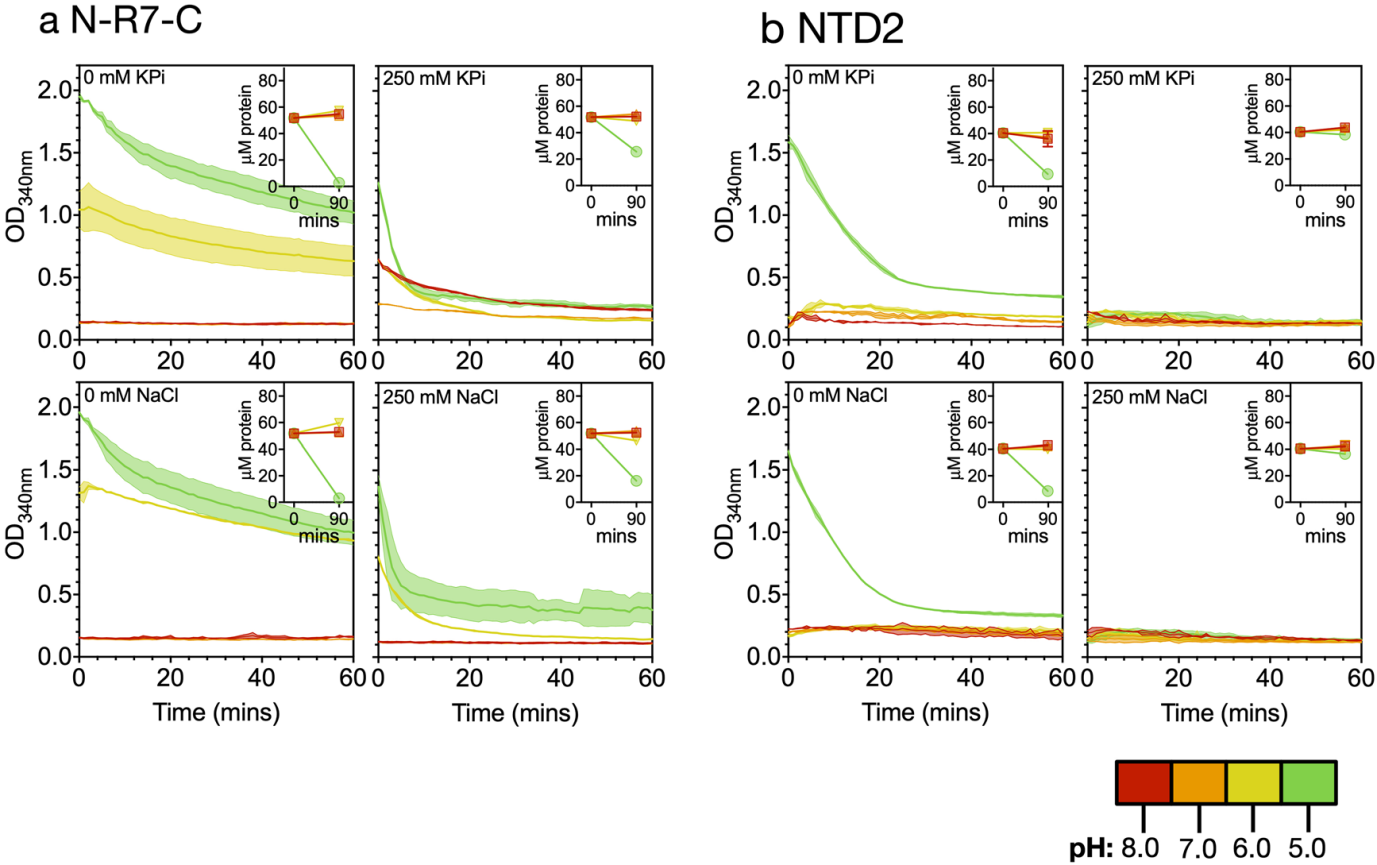
Changes in turbidity and soluble protein concentration for NTD2 and N-R_7_-C in response to pH and salt. Turbidity as measured by OD_340nm_ (left axis) over time at various pH values and salt concentrations. pH is indicated by colour: pH 5 (green), pH 6 (yellow), pH 7 (orange), pH 8 (red), as also indicated in the legend. The right axis shows protein concentration, as determined by nanodrop at OD_280nm_ before and after the assay, shown by pH 8: squares, pH 7: upwards triangles, pH 6: downwards triangles and pH 5: circles. Error bars show the standard deviation of three replicates in both cases. Error bars show the standard deviation of three technical replicates.

We wished to further investigate the effects of pH and salt on the constituent domains of N-R_7_-C, by measuring turbidity and soluble protein concentration over time in response to different combinations of pH and salt (Fig. 6, Supplementary Fig. S10). Potassium phosphate was prepared at each pH from solutions of KH_2_PO_4_ and K_2_HPO_4_, referred to as KPi here. Where aggregation occurred, an initial fast increase in turbidity was observed, followed by differing rates of decreasing turbidity over time. A rise and subsequent fall in turbidity generally led to substantially decreased protein quantification, suggesting the fall in turbidity was not due to proteins going back into solution. Other work investigating natural spider silk dopes has suggested this phenomenon might be due to the formation of larger aggregates, causing a loss in light scattering^58^. However, an alternative hypothesis is that the aggregates are becoming stuck to the walls of the 96-well plate.

NTD2 aggregated in the absence of NaCl or KPi at pH 5.0 (Fig. 6). Dimerization of spidroin N-terminal domains is induced via a protonation-induced dipole interaction^59^. In the absence of salt, our results suggest that at pH 5.0 the stabilisation of like-charge clusters on the protein surface by electrolytes is essential to prevent undesirable aggregation, as has been previously been suggested^59^. Interestingly, the addition of NaCl has also been shown to shift the pKa for dimerization towards a more acidic pH^60^.

Furthermore, proteins featuring NTD2 showed a negative shift in thermo-stability at pH 5.0 in the absence of salt, as determined by differential scanning fluorimetry (Supplementary Fig. S11). However, many simultaneous changes are likely responsible for the observed derivative plots, and in some cases no peak was observed, making analysis difficult.

Overall, our results suggest that assembly of mini-spidroins featuring an NTD in the absence of salt, may be via aggregation at the NTD rather than through NTD dimerization. Assembly in this way is likely undesirable for a biomimetic spinning process. Crucially, NTD has been shown to retain a dimeric structure in fibers^61^, suggesting correct dimerization rather aggregation is important^25^. Further experiments are necessary to pick apart the multiple simultaneous changes likely responsible for the observed turbidity response for the complete mini-spidroin N-R_7_-C (Fig. 6). However, NTD1 and CTD1 have recently been shown not to interact in solution^29^, which may well be the case for NTD2 and CTD1 and would suggest data on the individual domains alone may offer useful insight into the complete mini-spidroins (Supplementary Fig. S10).

### Biomimetic wet spinning of a complete mini-spidroin results in synthetic spider silk fibers

**Figure 7:**
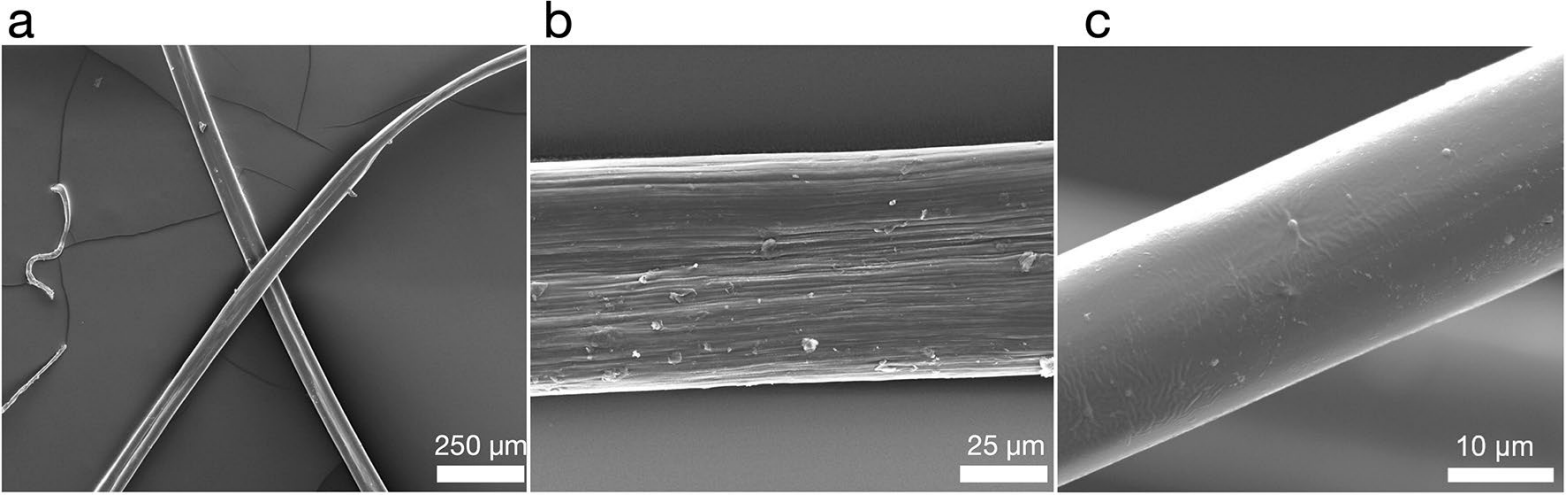
SEM images of spun fibers of N-R_7_-C. Scale bars show 250 μM (**a**), 25 μm (**b**) 10 μm (**c**). Surface striations are observed at high magnification in some cases (**b**), but not others (**c**). The fiber shown in panel (**c**) represents a second batch of fibers, spun on a separate occasion to the fibers shown in (**a**) and (**b**).

**Figure 8:**
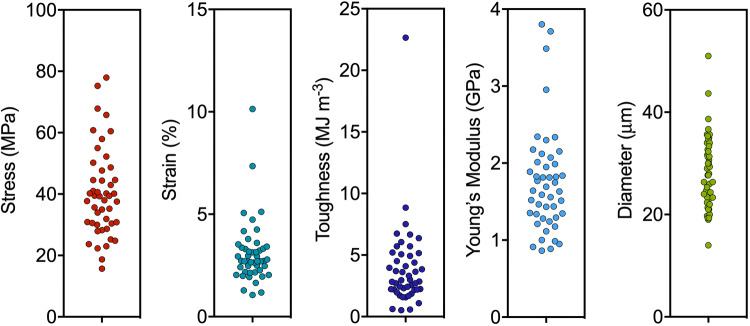
Mechanical testing of synthetic spider silk fibers from N-R_7_-C. Mechanical properties were calculated from stress strain curves (Supplementary Fig. S11).

**Figure 9:**
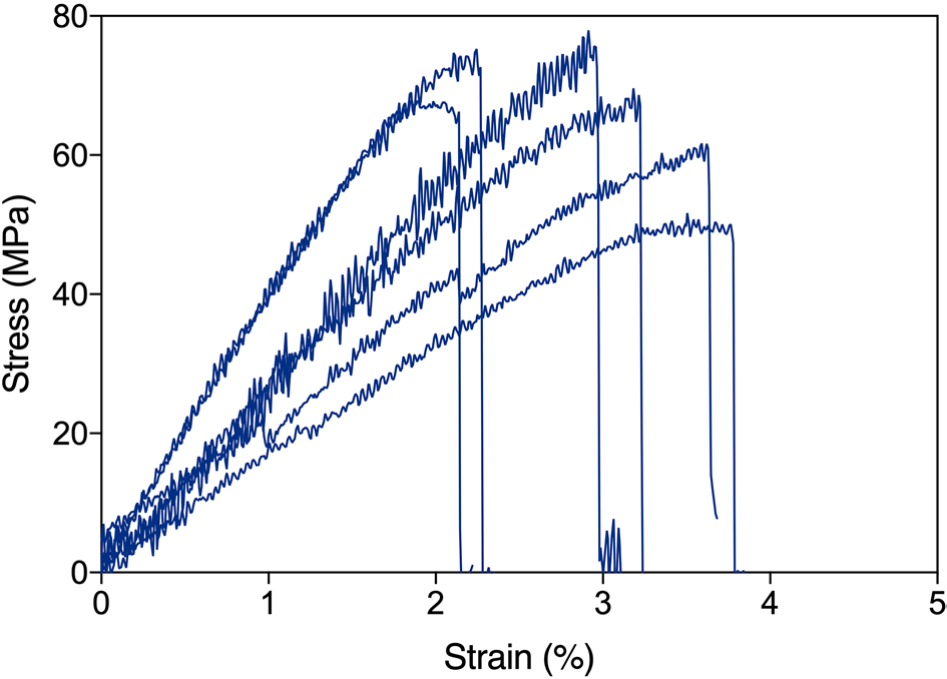
Stress strain curves for a selection of the best fibers tested. All experiments carried out on the same day with environmental conditions of 28 °C, 52% humidity. Fibers were mounted on cardboard frames and mounted into a tensile testing machine (Instron 3344; Instron Ltd.), equipped with a 10 N load cell. Tensile tests were performed at a rate of 0.5 mm/min. Mechanical properties calculated using diameters determined by light microscopy.

The production of fibers from N-R_7_-C by the extrusion into a biomimetic coagulation bath was tested. A coagulation bath consisting of 50 mM sodium acetate, 500 mM potassium phosphate pH 5.0 was used, inducing a pH drop in combination with salting-out. An aggregated mass formed at the tip of needle from which a fiber could be pulled by tweezers and collected continuously onto a rotating collector (Supplementary Material 2-movie). This is analogous to how spiders spin silk, with the silk pulled from the spinneret rather than pushed, in a process referred to as pultrusion^62^. This use of mechanical force is also important in achieving strong, aligned fibers, evidenced by the need for post-spin drawing in many examples of synthetic spider silk spinning^42,63^. The diameters of the fibers, as determined by light microscopy, assuming a circular cross section, varied between 14 and 51 μm (Fig. 7, Supplementary Fig. S12). Engineering stress and strain of as-spun fibers were determined by tensile testing, with a mean ultimate tensile strength of 40.3 MPa, and a maximum of 78 MPa (Fig. 8). The fibers showed on average a lower Young’s modulus than native spider silk (1.8 ± 0.7 compared to 11.6 ± 0.7 GPa for native spider silk^64^). Thinner fibers correlated with higher ultimate tensile strength (UTS), and higher values for Young’s modulus (R = − 0.62 and R = − 0.53 respectively, Supplementary Figs. S13 and S14, where R corresponds to the correlation coefficient). Previous analysis has shown that thinner fibers are typically stronger, and that there is a strong linear correlation between the square root of modulus/diameter, and tensile strength^65^. Our data also follow this trend (Supplementary Fig. S14, R = 0.72). A comparison with the mechanical properties obtained in other studies is shown in Table 1. We also direct the reader to recent reviews for further examples of other artificial silk spinning for comparison^6,63^. Six of the best fibers in terms of their ultimate tensile strength are shown in Fig. 9. A total of 47 fibers were tested (Fig. 8).

**Table 1:** Comparison of mechanical properties.

	N-R_7_-C (this study)	Native Dragline silk *A. trifasciata*	NT2RepCT	N1L(AQ)24NR3	Synthetic 192-mer	eTuSp1	NT-MaSp1s-CT	MaSp1 (WS-PSD-3x)	eMaSp1s
References	This study	^64^	^9^	^13^	^73^	^41^	^30^	^36^	^42^
Diameter (μm)	28 ± 7	~ 3	12 ± 2	20 ± 6	5.7 ± 1.3	–	92 ± 5	–	49 ± 7
Extensibility (%)	3 ± 1.5	17 ± 0.04	37 ± 5	54 ± 15	22 ± 8	17.5 ± 1.6	97 ± 2	18.3 ± 12.8	102 ± 24
Strength (MPa)	40 ± 14	890 ± 130	162 ± 8	308 ± 131	1,031 ± 111	78.3 ± 4.2	52.92	286.2 ± 137.7	282 ± 66
Toughness (MJ/m^3^)	3.9 ± 3.4	100 ± 40	45 ± 7	90 ± 29	114 ± 58	11.5 ± 0.8	3.56 ± 0.1	37.7 ± 28.8	144 ± 44
Young's modulus (GPa)	1.8 ± 0.7	11.6 ± 0.7	6 ± 0.8	5 ± 2	13.7 ± 3	2.4 ± 0.4	1.65 ± 0.3	8.4 ± 4.3	1.5 ± 0.3

The best examples of the mechanical properties for fibers spun from recent recombinant spidroins, as reported in the referenced articles. Data for N-R7-C is reported with the standard deviation for all recorded fibers (Fig. 8 and Supplementary Fig. S11).

Wide-angle X-ray diffraction (WAXD) was conducted on a bundle of fibers to probe their crystallinity (Supplementary Fig. S15). A broad amorphous region was observed along with two distinguishable diffraction peaks at approximately 10.4 and 22.6 degrees, indexed respectively as the (100) and (120) Bragg reflections of β-sheet crystallites as reported by Du et al.^66^. The average β-sheet crystallite size was calculated as 4.8 nm × 2.0 nm by application of the Scherrer equation on the deconvoluted peaks; L = Kλ/βcosθ where L is the mean size of the crystallite domains, λ is the X-ray wavelength (1.542 Å), K is the dimensionless shape factor (taken as 0.9), β is the peak full width at half maximum (FWHM) and θ is the diffraction angle. The data were broadly in agreement with WAXD patterns of natural spider silk reported by Du et al.^66^.

Our results are in good agreement with previous work^9^, which demonstrated the wet-spinning of a mini-spidroin with both terminal domains via a pH drop. We selected a pH drop to 5.0 for comparison with this work, and have more thoroughly investigated the effects of ionic strength on this spinning process, demonstrating this to be an important factor in this approach. The development of a spinning process utilising multiple smaller pH drops, or a continuous gradient could offer further improvements^3,30,36,41,42,67^. In the development of such an approach it would likely be insightful to further consider the isoelectric point (pI) of the mini-spidroins and their constituent domains^57^. The use of microfluidic chips also offers a promising avenue for improved spinning techniques^36^.

## Conclusions

Biomimetic spinning offers a number of advantages over spinning using denaturing conditions, which arguably produces aggregates of denatured protein rather than correctly assembled spider silk. However, in order to be effective, we must understand the impact of pH, ion exchange and shear stress on biomimetic spinning dopes consisting of “complete” spidroins featuring both terminal domains. This work examines the production of a range of such mini-spidroins, and thoroughly investigates the conditions necessary for their biomimetic spinning. The shear thinning observed for our small complete mini-spidroin is an attractive property from a manufacturing perspective, offering superior wet-spinnability. The terminal domains allow polymerisation of a spidroin upon a pH drop. However, our results suggest that a pH drop alone is insufficient and a combination with salting-out, for which spiders use the exchange of sodium and chloride ions for potassium and phosphate ions, is critical for the production of robust fibers in a biomimetic wet spinning process. Using these conditions, biomimetic wet spinning allowed fibers to be spun continuously from a small mini-spidroin, with fibers formed from many aligned fibrils. However, fibers with diameters larger than native spider silk, and relatively poor mechanical properties, suggest an improved biomimetic spinning process is required. Our results provide further insight for development of such a biomimetic spinning technique, which might combine shear with a biomimetic assembly buffer, as characterized here. Such a technique could offer substantial improvements to the quality of fibers achievable from small mini-spidroins, which are attractive for production at industrial scale since they can be produced at high yields. Future investigations into the underlying mechanisms by which ionic strength and pH drop facilitate fiber formation, could offer further improvements.

## Methods

### Spidroin cloning, protein expression and purification

Genes for the N- and C-terminal domains (NTD1, NTD2, CTD1 and CTD2) were synthesised and cloned into the NcoI and HindIII restriction sites of the plasmid pNIC28-BSA4^68^. Protein sequences were based on GenBank accession numbers EF595246 and EF59524^69^. The plasmid designated pTE1253 (NTD2-BsaI-CTD1-pNIC28) was generated by Gibson assembly^70^. R_18_ was gene synthesised with the inclusion of BsaI and BpiI restriction sites. Repetitive regions below R_18_ in size were generated by PCR with the inclusion of BsaI and BpiI restriction sites, and cloned directly into pTE1253 (Supplementary Fig. S4). Repetitive regions larger than R_18_ were generated according the scheme outlined in Supplementary Fig. S3, before sub cloning into pTE1253. All constructs featured an N-terminal 6 × His tag. Sequences were confirmed by restriction digest and Sanger sequencing. Where sequences were too large and repetitive for Sanger sequencing, only the beginning and end of the sequence was confirmed. Plasmid sequences are available as supplementary material (Supplementary Material 3 zip). Protein sequences are available at the end of the Supplementary Material as Supplementary Sequences 1. Cloning was carried out in *E. coli* 5α.

Protein expression was carried out in *E. coli* BL21(DE3) in Terrific Broth media with the addition of 100 μg/μL kanamycin. Cells were grown to approximately 0.8 OD_600nm_ (optical density at 600 nm) at 37 °C with shaking at 180 rpm, at which point IPTG was added to a concentration of 200 μM. Temperature was dropped to 20 °C for protein expression overnight. Cell lysate was prepared by sonication on ice followed by centrifugation to remove the insoluble fraction. Proteins were purified from cell lysate by immobilized metal affinity chromatography using a Ni–NTA resin, eluted using 250 mM imidazole. Purified protein was dialyzed twice against 25 mM TrisHCl pH 8.0, at 4 °C. Aggregated protein following dialysis was removed by centrifugation. Protein expression and purification was analyzed by SDS-PAGE. Protein concentrations were determined in triplicate by OD_280nm_ using a Nanodrop 2000 (Thermo Scientific), using an extinction coefficient and molecular weight for each protein calculated using the ExPaSy ProtParam tool^71^. Where necessary, dilutions of proteins were made before determining the concentrations. Single use aliquots of protein were stored at − 80 °C where appropriate.

### Size exclusion chromatography of NTD2

Buffers consisting of 25 mM Tris pH 8.0 with either 0 or 300 mM NaCl, and 25 mM MES pH 5.5 with either 0 or 300 mM NaCl, were prepared and filtered. 250 μL of purified NTD2 at 3.4 mg/mL was loaded onto a Superdex 200 Increase 10/300 GL size exclusion column pre-equilibrated in the relevant buffer, using an AKTA Pure system. The sample was eluted over 1.2 column volumes, with samples coming off the column monitored at OD_280nm_. Retention times were compared to a standard curve of known proteins, and the theoretical molecular weight of NTD2 to estimate oligomeric state.

### Tryptophan fluorescence

Tryptophan fluorescence for NTD2 at 0.8 mg/mL was recorded between 310 and 400 nm (bandwidth 10 nm) while exciting at 280 nm (bandwidth 20 nm). The assay was performed in triplicate in a 96-well microtiter plate using a M200 Infinite plate reader (Tecan). pH values were achieved using the following buffers at a final concentration of 50 mM. MES: pH 5.5, 5.7, 5.9, 6.1, 6.3, 6.5 and 6.8. MOPS: pH 6.5, 6.7, 6.9, 7.1, 7.3, 7.5 and 7.7. HEPES: pH 6.8, 7.0, 7.2, 7.4, 7.6, 7.8, 8.0 and 8.2. Blank measurements were recorded in every condition and subtracted from the final reading.

### Dynamic scanning fluorimetry

Dynamic scanning fluorimetry assays were carried out using 96-well PCR plates (BioRad) in a CFX Connect Real-Time PCR detection system (BioRad), using the HEX filter^32–34^. Assay volume was set at 30 μL Suitable protein concentrations between 1 and 10 μM for each assay were determined by titrating the amount of protein. A master mix of protein and SYPRO Orange was prepared, and 5 μL added to every well. SYPRO Orange was used at an assay concentration of 5×. 25 μL of each assay buffer solutions were transferred by multi-channel pipette to the assay plate in triplicate. The assay plate was sealed and briefly centrifuged before starting the assay. After a 3 min hold at 25 °C, temperature was increased by 0.4 °C every 30 s up to 95 °C. Peaks in dF/dt, corresponding to the T_M_ of the protein, were identified using the Biorad CFX Manager software.

To investigate the response of CTD1 to pH, a range of pH values between 8.2 and 5.5 were set up in a 96 deep-well block. pH values were achieved using the following buffers at a final concentration of 50 mM. MES: pH 5.5, 5.7, 5.9, 6.1, 6.3, 6.5 and 6.8. MOPS: pH 6.5, 6.7, 6.9, 7.1, 7.3, 7.5 and 7.7. HEPES: pH 6.8, 7.0, 7.2, 7.4, 7.6, 7.8, 8.0 and 8.2. No salt, or assay concentrations of 250 mM NaCl or 250 mM KCl were added to three separate sets of buffers. Subsequent assays used assay concentrations of 40 mM HEPES (pH 8.0 and 7.0), 40 mM MES (pH 6.0), and 40 mM sodium acetate (pH 5.0). NaCl or KPi concentrations were added at each pH for assay concentrations between 0 and 400 mM. Potassium phosphate was prepared at each pH from solutions of KH_2_PO_4_ and K_2_HPO_4_, referred to as KPi here.

### Rheology flow sweeps

Rheology was conducted using 180 μL sample of 200 mg/mL the mini-spidroin designated N-R_7_-C for shear sweep measurements, or 100 mg/mL N-R_7_-C for the frequency, amplitude and time sweeps. The lower concentration of 100 mg/mL was used initially to facilitate more preliminary experiments. A discovery HR-2 hybrid rheometer (TA Instruments) was used, with a parallel plate geometry with a plate diameter of 20 mm. A geometry gap of 500 μm was used and a solvent trap attached to prevent evaporation. Experiments were carried out at 25 °C. The viscosities of the solutions under shear sweeps were investigated using a logarithmic steady shear rate increase from 0.01 to 1,000 1/s. Ten datapoints were collected per decade. An equilibration time of 5 s and averaging time of 30 s was used. The control rate settings were set to have the motor in ‘Auto’ mode. Samples were run four times each, with and without a 15 min settle time to check for the effect of immediate consecutive runs on samples' viscosity.

### Turbidity assays to investigate aggregation

An array of buffer conditions were prepared in a 96-well microtitre plate. Assay concentrations of 50 mM HEPES (pH 8.0 and 7.0), 50 mM MES (pH 6.0), or 50 mM sodium acetate (pH 5.0) were prepared, each with 0–250 mM assay concentrations of NaCl or KPi, in triplicate. KPi stock solutions were prepared at each pH from KH_2_PO_4_ and K_2_HPO_4_. Assays were initiated by the addition of 25 μL protein for an assay concentration of approximately 40 μM, gently mixed by tapping the plate before placing into the Clariostar plate reader (BMG Labtech). To measure changes in turbidity, the average of four OD_340_ readings was recorded every minute per well for one hour. Protein concentrations before and after the assay were determined by taking 2 μL of protein from the top of each well and measuring OD_280_ using a nanodrop.

### Analysis of mini-spidroin assembly

To investigate the effect of potassium phosphate during a wet spinning process, buffers containing 50 mM HEPES pH 8.0, or 50 mM sodium acetate pH 5.0 were prepared with the addition of 0 to 500 mM potassium phosphate, prepared from KH_2_PO_4_ and K_2_HPO_4_ at the relevant pH. N-R_7_-C at 100 mg/mL was extruded at 0.5 mL/h using a syringe pump (Cole-Palmer 74,900 series) with a 1 mL syringe, through a blunted 16G needle.

### Fiber spinning and analysis

N-R_7_-C at 300 mg/mL was extruded using a syringe pump through a pre-pulled glass capillary (MGM-3-1.5-5NF, 30 μm tip, FivePhoton Biochemicals) at 0.5 mL/h using a syringe pump (Cole-Palmer 74,900 series) with a 1 mL syringe into a coagulation bath of 50 mM sodium acetate, 500 mM potassium phosphate pH 5.0. A fiber was pulled using tweezers onto a custom-made rotating collector onto which fibers were collected continuously at approximately 9.6 m/min.

Individual fibers were mounted onto cardboard mechanical testing windows with a 5 mm gauge length using scotch tape. Each fiber on a window was imaged by light microscopy (Leica DMI6000) at 20 × magnification. Fiber diameters were measured using ImageJ at multiple points along the fiber, and the average taken. The cross sectional area of each fiber was calculated from the average diameter, for subsequent mechanical testing measurements. Fibers on cardboard frames were mounted into a tensile testing machine (Instron 3344; Instron Ltd.), equipped with a 10 N load cell. Upon mounting, the sides of the window were cut and the fiber loaded. Tensile tests were performed at a rate of 0.5 mm/min at room temperature and humidity (28 °C, 52% humidity). Engineering stress was calculated from the measured load using the calculated cross-sectional area. The ultimate tensile stress (UTS) and strain to failure were determined, Young’s modulus was calculated from initial linear portion of the stress–strain curve, and toughness calculated from the area under the stress–strain curve.

Fiber morphologies were assessed using scanning electron microscopy (SEM). Fibers were mounted onto aluminium SEM studs with double-sided conductive carbon tape and sputter-coated with gold/palladium (10 nm thickness) (Gatan Model 682 Precision Etching Coating System, USA). Fibers were imaged using a Hitachi S300 N SEM and an FEI Quanta 250 FEG-SEM.

Fiber crystallinity was determined by wide-angle X-ray diffraction (WAXD) using a PANalytical X’Pert Pro (UK) instrument with Kα radiation source (Kαav = 1.542 Å). A 1D detector was employed meaning 2D WAXD images could not be produced. The fiber bundle was mechanically attached to a zero-background holder and the diffraction angle ranged between 5° and 60° with a scanning rate of 1°/min.

## Supplementary information

Supplementary information 1.

Supplementary information 2.

Supplementary information 3.
