# Supplementary material for: The effect of terminal globular domains on the response of recombinant mini-spidroins to fiber spinning triggers
Source: Sci Rep. 2020 Jun 30;10:10671. doi: 10.1038/s41598-020-67703-1 (PMC7327021; doi:10.1038/s41598-020-67703-1)
Supplement: Supplementary file 1 — Supplementary information 1. [file 41598_2020_67703_MOESM1_ESM.docx]

**The effect of terminal globular domains on the response of recombinant mini-spidroins to fiber spinning triggers**

William Finnigan^1^, Aled D. Roberts^1^, Cosimo Ligorio^2^, Nigel S. Scrutton^1^, Rainer Breitling^1^, Jonny J. Blaker^3^ and Eriko Takano^1*^

^1.^ Manchester Institute of Biotechnology, Manchester Synthetic Biology Research Centre SYBIOCHEM, Department of Chemistry, The University of Manchester, Manchester, M1 7DN, UK

^2.^ Manchester Institute of Biotechnology, Department of Materials, The University of Manchester, Manchester, M1 7DN, UK

^3.^ Bio-Active Materials Group, Department of Materials, The University of Manchester, Manchester, M13 9PL, UK

# SUPPLEMENTARY FIGURES

**Figure S1 – NTD and CTD expression and purification**

Samples of the soluble fraction (Sol), insoluble fraction (Insol), flow-through (FT) and elution (E) following nickel affinity chromatography of E. coli cell lysate, after expression NTD1, NTD2, CTD1 and CTD2 domains analysed by SDS-PAGE. Over-expression of the proteins is indicated at the expected molecular weight by the red arrows in each case. All proteins were successfully purified via an N-terminal 6xHis tag, as shown in the lanes labelled P. Markers to the left of each gel are shown in kDa.

1. Expression and purification of NTD1, NTD2, CTD1 and CTD2. Initially part of the spidroin repetitive sequence was cloned together with the NTDs resulting in larger domains, due to misannotation of this extra sequence as part of the NTD in previous work ^1^. NTD1 shows proteolysis in the soluble fraction and following purification. Similar proteolysis has been shown previously for a spidroin N-terminal domain from the nursery-web spider *Euprosthenops australis* ^2^.
2. Expression of NTD1 and NTD2 with the excess repetitive sequence removed, resulting in a smaller purified domain. The smaller version of NTD2 was used in subsequent work. While NTD1 no longer shows proteolysis, protein aggregates were observed following purification. For this reason NTD2 was chosen for characterisation and for use in mini-spidroins.

**Figure S2 –** Differential scanning fluorimetry of CTD1 at various pH values (pH 8.2 (red) to pH 5.5 (green), with 250 mM NaCl. The plot shows the derivative of the fluorescence signal, with the peak corresponding to the denaturing temperature (TM) of the protein. (The assay was also performed with 250 mM KCl instead of NaCl, giving similar results.)

**Figure S3** **– Initial fluorescence of CTD1 in DSF assay.**

SYPRO Orange fluorescence upon binding to hydrophobic regions of proteins. A higher initial fluorescence at lower pH suggests more exposed hydrophobic regions of CTD1 at these pH values. The assay was performed in the presence of 250 mM NaCl and KPO_4_, and without salt as indicated. Error bars show the standard deviation of three technical replicates.

**Figure S4 – TypeIIS cloning scheme for pseudo-scarless duplication of repetitive sequences**

Scheme shows the use of restriction enzymes which can be used to carry out pseudo-scarless duplication of repetitive regions. This is achieved by designing the cut sites of the type IIS restriction enzymes (BsaI and BpiI), to cut at a sequence coding for an alanine and a glycine, which will be present at the position regardless. The figure shows: **1**. An initial plasmid with a single repeating unit and kanamycin resistance. **2.** Digestion of the plasmid in two separate reactions with either EcoRI and BpiI, or BsaI and BamHI. The digestion results in two fragments, which can be ligated together with a second plasmid with chloramphenicol resistance, which has been cut with EcoRI and BamHI. **3.** The ligation of these three fragments results in a new plasmid with the same restriction sites as the first, but with the repeating unit now duplicated. The scar site from the duplication codes for alanine and glycine in frame with the rest of the coding sequence.

**Figure S5 – pTE1253 (NTD2-BsaI-CTD1-pNIC28) mini-spidroin expression vector.**

1. Plasmid map of pTE1253. A dashed box indicates the relevant section for mini-spidroin expression, shown in B.
2. Region for cloning repetitive sections into the BsaI sites, for the construction of mini-spidroins with both terminals.

**Figure S6 – Expression of mini-spidroin constructs after four hours expression.** Two independent SDS-PAGE of soluble (S) and insoluble (I) fractions of *E. coli* lysate following expression of various mini-spidroins for four hours at 20°C. Overexpressed proteins at the expected molecular weight are indicted by red arrows. Where no protein is detected the expected size is indicated by the red boxes. Markers to the left of each gel are shown in kDa.

**Figure S7 – Ni IMAC purification of N-R_7_-C mini-spidroin.**

Samples of the soluble fraction (Sol), insoluble fraction (Insol), flow-through (FT) and elution (Purified) following nickel affinity chromatography of *E. coli* cell lysate, after expression of the mini-spidroin. Markers to the left the gel are shown in kDa.

**Figure S8 – Preliminary frequency, amplitude and time sweeps of N-R_7_-C at 100 mg/mL.** Fixed parameters are displayed below each figure. The storage modulus (G’), and loss modulus (G’’) was recorded in each case. **A.** Frequency sweep suggests that the sample behaves like a weak gel, as G’ is greater than G’’. **B.** Amplitude sweep also suggests the sample behaves as a weak gel, as G’ is greater than G’’. The weak gel appears to break at approximately 400 % strain. **C.** Time sweep was conducted directly after the amplitude sweep with the same sample, and shows no memory of the gel breaking.

**Figure S9 – Multiple flow sweeps of 200 mg/mL (20 % w/v) N-R_7_-C.** Two samples were tested with multiple consecutive runs, sample A – red, sample B – blue. Consecutive runs are shown in order of circles, squares, upwards triangle, downwards triangle. Run two for sample B showed very noisy viscosity readings and so was not included. Shear history did not appear to have an effect on the rheology of the sample. **A.** Viscosity in response to shear. **B.** Normal force in response to shear.

**Figure S10** **NTD2 (A), CTD1 (B), N-R_7_-C (C), and NC (D) characterization** The effect of pH and salt. Turbidity as measured by OD_340nm_ (left axis) over time at various pH values and salt concentrations. pH is indicated by colour: pH 5 (green), pH 6 (yellow), pH 7 (orange), pH 8 (red), as also indicated in the legend. The right axis shows protein concentration, as determined by nanodrop at OD_280nm_ before and after the assay, shown by pH 8: squares, pH 7: upwards triangles, pH 6: downwards triangles and pH 5: circles. Error bars show the standard deviation of three replicates in both cases.

**Figure S11 – DSF of NTD2, CTD1, NC and N-R_7_-C at different pH’s and salt concentrations** Three replicates are plotted for each condition. Temperature is shown in ^o^C. NaCl or KPi concentrations are indicated according to the colour chart. A shift in TM is observed at pH 5.0 for N-R7-C, NC or NTD2 in the absence of NaCl or KPO_4_, visible either as a distinct peak or as a shoulder towards a lower TM. This shift is not visible for CTD1, suggesting the response is caused by the presence of NTD2 in N-R7-C and NC.

**Figure S12.** A representative polarised light microscopy image of a fiber spun from N-R_7_-C.

**Figure S13 – Stress strain curves for tensile tests of N-R_7_-C fibers.** All experiments carried out on the same day with environmental conditions of 28 ^o^C, 52 % humidity. Fibers were mounted on cardboard frames and mounted into a tensile testing machine (Instron 3344; Instron Ltd.), equipped with a 10 N load cell. Tensile tests were performed at a rate of 0.5 mm/min. Mechanical properties calculated using diameters determined using light microscopy.


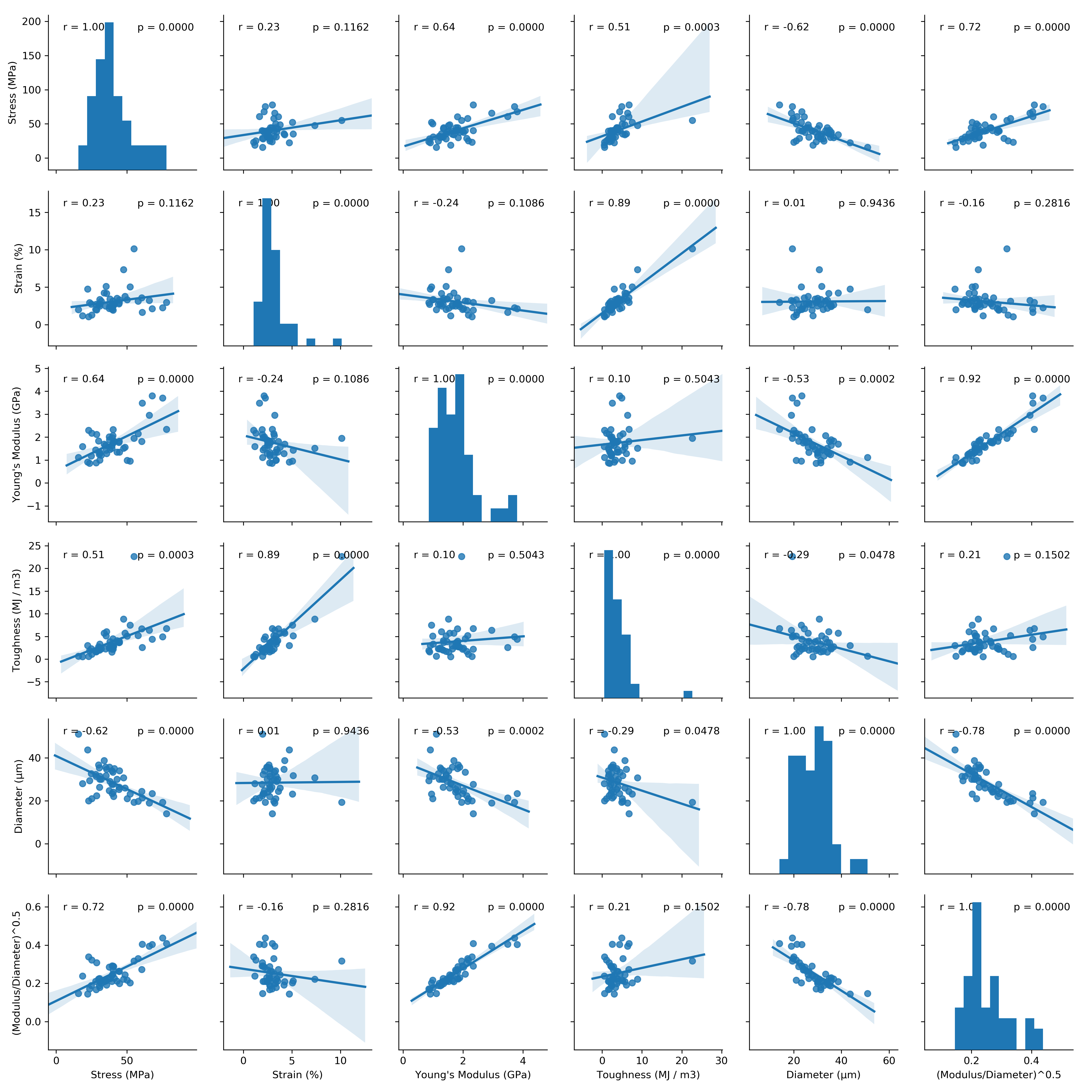


**Figure S14 – Correlation matrix of mechanical properties for fibers spun from N-R7-C**. Generated using the python package Seaborn. Correlation coefficient (r), and significance level (p) for each pair of factors is shown. Histograms showing the distribution of each factor are shown on the diagonal. Error bars show the 95 % confidence interval for the linear regression.


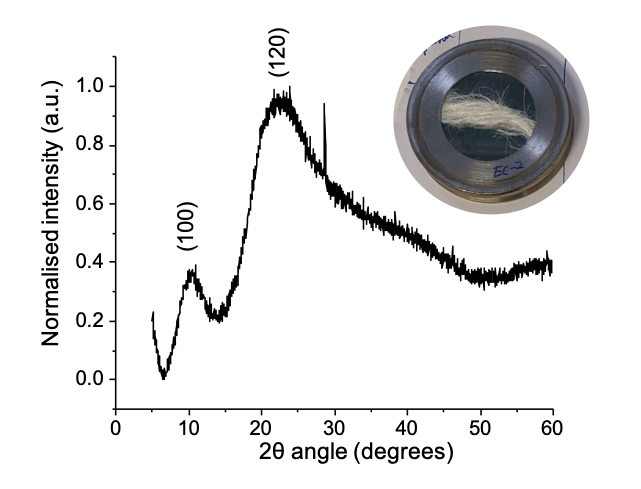


**Figure S15.** Wide-angle X-ray diffraction (WAXD) pattern of a bundle of fibers spun from spun from N-R7-C. **Inset:** fiber bundle on sample holder.

| >NTD2_ unstructured_repetitive_region  MHHHHHHSSGVDLGTENLYFQSMALGQANTPWSSKENADAFIGAFMNAASQSGAFSSDQIDDMSVISNTLMAAMDNMGGRITQSKLQALDMAFASSVAEIAVADGQNVGAATNAISDALRSAFYQTTGVVNNQFITGISSLIGMFAQVSGNEVSYSSAGSSSAAASEAVSAGQGPAAQPVYAPSGASAAAAAASGAAPAIQQAYERGGSGSAAAAA* |
| --- |
| >NTD2  MHHHHHHSSGVDLGTENLYFQSMALGQANTPWSSKENADAFIGAFMNAASQSGAFSSDQIDDMSVISNTLMAAMDNMGGRITQSKLQALDMAFASSVAEIAVADGQNVGAATNAISDALRSAFYQTTGVVNNQFITGISSLIGMFAQVSGNEV* |
| >CTD1  MHHHHHHSSGVDLGTENLYFQSMGSGPGQIYYGPQSVAAPAAAAASALAAPATSARISSHASALLSNGPTNPASISNVISNAVSQISSSNPGASACDVLVQALLELVTALLTIIGSSNIGSVNYDSSGQYAQVVTQSVQNAFA* |
| >NC  MHHHHHHSSGVDLGTENLYFQSMALGQANTPWSSKENADAFIGAFMNAASQSGAFSSDQIDDMSVISNTLMAAMDNMGGRITQSKLQALDMAFASSVAEIAVADGQNVGAATNAISDALRSAFYQTTGVVNNQFITGISSLIGMFAQVSGNEVGSGAGGGSGPGQIYYGPQSVAAPAAAAASALAAPATSARISSHASALLSNGPTNPASISNVISNAVSQISSSNPGASACDVLVQALLELVTALLTIIGSSNIGSVNYDSSGQYAQVVTQSVQNAFAGS* |
| >N-R7-C  MHHHHHHSSGVDLGTENLYFQSMALGQANTPWSSKENADAFIGAFMNAASQSGAFSSDQIDDMSVISNTLMAAMDNMGGRITQSKLQALDMAFASSVAEIAVADGQNVGAATNAISDALRSAFYQTTGVVNNQFITGISSLIGMFAQVSGNEVAGGGAGQGGQGGYGRGGYGQGGAGQGGAGAAAAAAAAGGAGQGGQGGYGQGGYGQGGAGQGGAAAAAAAAAGGAGQGGYGRGGAGQGGAGGSGPGQIYYGPQSVAAPAAAAASALAAPATSARISSHASALLSNGPTNPASISNVISNAVSQISSSNPGASACDVLVQALLELVTALLTIIGSSNIGSVNYDSSGQYAQVVTQSVQNAFAGS* |
| >N-R10-C  MHHHHHHSSGVDLGTENLYFQSMALGQANTPWSSKENADAFIGAFMNAASQSGAFSSDQIDDMSVISNTLMAAMDNMGGRITQSKLQALDMAFASSVAEIAVADGQNVGAATNAISDALRSAFYQTTGVVNNQFITGISSLIGMFAQVSGNEVAGGGAGQGGQGGYGRGGYGQGGAGQGGAGAAAAAAAAGGAGQGGQGGYGQGGYGQGGAGQGGAAAAAAAAAGGAGQGGYGRGGAGQGGAAAAAAAAGAGQGGYGGQGAGQGGAGAAAAAAAAGGSGPGQIYYGPQSVAAPAAAAASALAAPATSARISSHASALLSNGPTNPASISNVISNAVSQISSSNPGASACDVLVQALLELVTALLTIIGSSNIGSVNYDSSGQYAQVVTQSVQNAFAGS* |
| >N-R13-C  MHHHHHHSSGVDLGTENLYFQSMALGQANTPWSSKENADAFIGAFMNAASQSGAFSSDQIDDMSVISNTLMAAMDNMGGRITQSKLQALDMAFASSVAEIAVADGQNVGAATNAISDALRSAFYQTTGVVNNQFITGISSLIGMFAQVSGNEVAGGGAGQGGYGRGGAGQGGAAAAGAGQGGYGGQGAGQGGAGAAAAAAAAGGAGQGGQGGYGRGGYGQGGAGQGGAGAAAAAAAAGGAGQGGQGGYGQGGYGQGGAGQGGAAAAAAAAAGGAGQGGYGRGGAGQGGAAAAAAAAGAGQGGYGGQGAGQGGAGAAAAAAAAGGSGPGQIYYGPQSVAAPAAAAASALAAPATSARISSHASALLSNGPTNPASISNVISNAVSQISSSNPGASACDVLVQALLELVTALLTIIGSSNIGSVNYDSSGQYAQVVTQSVQNAFAGS* |
| >N-R36-C  MHHHHHHSSGVDLGTENLYFQSMALGQANTPWSSKENADAFIGAFMNAASQSGAFSSDQIDDMSVISNTLMAAMDNMGGRITQSKLQALDMAFASSVAEIAVADGQNVGAATNAISDALRSAFYQTTGVVNNQFITGISSLIGMFAQVSGNEVAGGAGQGGQGGYGRGGYGQGGAGQGGAGAAAAAAAAGGAGQGGQGGYGQGGYGQGGAGQGGAAAAAAAAAGGAGQGGYGRGGAGQGGAAAAGAGQGGYGGQGAGQGGAGAAAAAAAAGGAGQGGQGGYGRGGYGQGGAGQGGAGAAAAAAAAGGAGQGGQGGYGQGGYGQGGAGQGGAAAAAAAAAGGAGQGGYGRGGAGQGGAAAAAAAAGAGQGGYGGQGAGQGGAGAAAAAAAAGGSGPGQIYYGPQSVAAPAAAAASALAAPATSARISSHASALLSNGPTNPASISNVISNAVSQISSSNPGASACDVLVQALLELVTALLTIIGSSNIGSVNYDSSGQYAQVVTQSVQNAFAGS* |
| Larger proteins are not shown, but available through the plasmid files in the supplementary information. |

Supplementary Sequences 1 – Protein sequences for a selection of proteins used in this study. Complete plasmid files are also available as an additional supplementary file.

**References**

1. Ayoub, N. A., Garb, J. E., Tinghitella, R. M., Collin, M. A. & Hayashi, C. Y. Blueprint for a High-Performance Biomaterial: Full-Length Spider Dragline Silk Genes. *PLoS One* **2**, (2007).

2. Hedhammar, M. *et al.* Structural properties of recombinant nonrepetitive and repetitive parts of major ampullate spidroin 1 from Euprosthenops australis: Implications for fiber formation. *Biochemistry* **47**, 3407–3417 (2008).
